# Evaluating different approaches to non-destructive nitrogen status diagnosis of rice using portable RapidSCAN active canopy sensor

**DOI:** 10.1038/s41598-017-14597-1

**Published:** 2017-10-26

**Authors:** Junjun Lu, Yuxin Miao, Wei Shi, Jingxin Li, Fei Yuan

**Affiliations:** 1 International Center for Agro-Informatics and Sustainable Development, College of Resources and Environmental Sciences, China Agricultural University, Beijing, 100193 China; 2 Department of Geography, Minnesota State University, Mankato, MN 56001 USA

## Abstract

RapidSCAN is a new portable active crop canopy sensor with three wavebands in red, red-edge, and near infrared spectral regions. The objective of this study was to determine the potential and practical approaches of using this sensor for non-destructive diagnosis of rice nitrogen (N) status. Sixteen plot experiments and ten on-farm experiments were conducted from 2014 to 2016 in Jiansanjiang Experiment Station of the China Agricultural University and Qixing Farm in Northeast China. Two mechanistic and three semi-empirical approaches using the sensor’s default vegetation indices, normalized difference vegetation index and normalized difference red edge, were evaluated in comparison with the top performing vegetation indices selected from 51 tested indices. The results indicated that the most practical and stable method of using the RapidSCAN sensor for rice N status diagnosis is to calculate N sufficiency index with the default vegetation indices and then to estimate N nutrition index non-destructively (R^2^ = 0.50–0.59). This semi-empirical approach achieved a diagnosis accuracy rate of 59–76%. The findings of this study will facilitate the application of the RapidSCAN active sensor for rice N status diagnosis across growth stages, cultivars and site-years, and thus contributing to precision N management for sustainable intensification of agriculture.

## Introduction

Nitrogen (N) inputs have made an important contribution to world food production^1^; however, over-application of N fertilizers has not given extra yields but has resulted in serious environmental problems in intensive agricultural, estuarine and coastal regions of China^2–7^. Precision N management (PNM) strategies matching N inputs with spatial and temporal crop N demands need to be developed for sustainable intensification of agriculture^8,9^. The development of fast, cost-effective and reliable methods for in-season site-specific diagnosis of crop N status is crucial for the success of wider applications of PNM strategies to improve N use efficiency and reduce negative environmental impacts^10,11^.

Active crop canopy sensors with their own light sources are not limited by environmental light conditions, therefore can be used any time of the day for non-destructive diagnosis of crop N status^10,12^. The GreenSeeker active canopy sensor with red (R) and near infrared (NIR) bands has been commonly used for N status diagnosis and PNM^11,13^. This sensor has two default vegetation indices (VIs): normalized difference vegetation index (NDVI) and ratio vegetation index (RVI). One challenge to using the GreenSeeker sensor is that NDVI has been found to saturate at medium to high biomass conditions, and thus may not be suitable for high yield cropping systems^12,14^. On the other hand, RVI’s representation of biomass is weak when the vegetation cover is sparse^15^, and RVI has a wide range of possible values that sometimes makes it difficult to interpret. Three waveband active canopy sensors like Crop Circle ACS 470 and Crop Circle ACS 430 that have a red edge (RE) band in addition to the R and NIR bands have been developed. It was found that these sensors with RE-based VIs were able to overcome the saturation problem of NDVI^12,14,16^. Compared with NDVI, the normalized difference red edge (NDRE) index as a widely used RE-based VI has been shown to be more resistant to the saturation problem^17^.

To facilitate practical applications, RapidSCAN CS-45, a light and convenient portable active crop canopy sensor with a built-in GPS, has been newly developed. It provides NDVI and NDRE as the default indices, in addition to the R, RE, and NIR waveband reflectance. Besides NDVI and NDRE, many different VIs can be calculated using the three spectral bands^18^. The key questions to be addressed are: How well can the default NDVI and NDRE be used to diagnose crop N status at key growth stages? How much improvement in crop N status diagnosis can be achieved using other VIs than NDVI and NDRE? Answering these questions can facilitate the application of this new sensor more effectively for in-season site-specific crop N status diagnosis and management.

A reliable N status indicator is N nutrition index (NNI), which is the ratio of measured plant N concentration (PNC) over critical PNC^19^. The critical PNC is the minimum PNC necessary to achieve maximum aboveground biomass (AGB) production^20^. It can be calculated using the critical N dilution curve, which describes the relationship between critical PNC and the AGB^19^. Different thresholds of NNI have been proposed in on-farm applications for diagnosing crop N status^10,19,21,22^. Due to the requirement of destructive sampling and laboratory analysis, direct on-farm measurement and application of NNI are limited.

By contrast, active canopy sensors provide multiple ways to non-destructively estimate crop NNI. One mechanistic approach is to use VIs obtained from crop sensors to estimate crop AGB and PNC. The critical PNC at a known AGB can be calculated using the critical N dilution curve^19,22^. The NNI can then be calculated accordingly. This approach can be termed as the NNI-PNC approach.

Similar to NNI-PNC, the second mechanistic approach is based on estimating plant N uptake (PNU) instead of PNC. The critical PNU is calculated by multiplying AGB and critical PNC. Consequently, NNI can be calculated using the ratio of estimated PNU over critical PNU. This approach is called NNI-PNU.

The third method, known as NNI-VI, is a semi-empirical approach, which uses crop sensor VIs to directly estimate NNI^10,18,23^. This NNI-VI approach is sensitive to crop phenological changes across growth stages; therefore, growth stage-specific models should be established^10,24,25^.

The next semi-empirical approach, termed NNI-NSI-VI or NNI-RI-VI, uses crop sensor VIs to calculate N sufficiency index (NSI) or response index (RI), which can then be used to estimate NNI^10,26^. Since the performance of crop sensors for N status diagnosis is influenced by many different factors other than N (e.g., seasonal variation, plant water status, diseases and pests, plant population, growth stages, and genotypes etc.), well-fertilized reference plots (WFRP) or strips have been used to normalize the sensor readings^27^. The NSI is a relative value calculated using sensor readings or VI values of the normal field or tested plots divided by the corresponding sensor data of WFRP^27^. The NSIs of chlorophyll meter readings were closely related to NNIs across years, cultivars, and growth stages^26,28^. Another normalization approach is to calculate RI, which is an inverse of NSI^29,30^, therefore named as NNI-RI-VI. Xia *et al*. found that RIs calculated using GreenSeeker NDVI or RVI performed consistently better than the original VIs for estimating spring maize NNI^10^.

The last semi-empirical approach, termed as NSI-VI or RI-VI, is to use the relationship between NSI (or RI) and NNI as well as the NNI threshold values to identify the corresponding NSI (or RI) thresholds and then use them to diagnose crop N status^10^. This is the only approach that does not need to estimate NNI.

Up to date, no study has been reported to evaluate the RapidSCAN CS-45 active canopy sensor for diagnosing crop N status by comparing these five different approaches. Therefore, this study took rice (*Oryza sativa* L.) as an example and the objectives were to (i) determine the potential of using the RapidSCAN sensor to diagnose rice N status non-destructively; (ii) identify the most suitable and practical approach from the five aforementioned ones for rice N status diagnosis using the RapidSCAN sensor.

## Results

### Variability of rice nitrogen status indicators

The N status indicators of rice varied greatly across different N rate treatments, management practices, cultivars, growth stages, sites, and years (Table 1). For the calibration dataset across all growth stages, the AGB was most variable, with a CV of 63.5%, followed by PNU (CV = 54.2%), PNC (CV = 26.6%) and NNI (CV = 19.4%). The average AGB and PNU increased with growth stages, whereas the average PNC decreased from 21.0 g kg^−1^ to 14.5 g kg^−1^. The validation dataset showed similar variability as the calibration one. In addition, more samples were collected from plot and on-farm experiments at the panicle initiation and stem elongation (PI&SE) growth stages as they are the key stages for panicle N fertilizer topdressing application in this region. The N status indicators at the PI&SE stages were more variable than those at the heading (HD) growth stage. The large variability of these parameters made it possible to evaluate the potential of using the RapidSCAN sensor for estimating and diagnosing rice N status.

**Table 1:** Descriptive statistics of rice aboveground biomass (AGB), plant nitrogen concentration (PNC), plant nitrogen uptake (PNU) and nitrogen nutrition index (NNI) for calibration and validation datasets across sites, cultivars, stages and years.

Dataset	AGB (t ha^−1^)			PNC (g kg^−1^)			PNU (kg ha^−1^)			NNI		
	Mean	SD	CV	Mean	SD	CV	Mean	SD	CV	Mean	SD	CV
Calibration dataset												
PI&SE (n = 286)	2.66	1.07	40.4	21.0	4.40	21.0	53.3	20.5	38.4	1.02	0.20	19.5
HD (n = 198)	8.77	1.57	17.9	14.5	2.29	15.8	128.3	33.8	26.3	1.09	0.20	18.5
Across all stages (n = 484)	5.16	3.28	63.5	18.3	4.87	26.6	84.0	45.6	54.2	1.05	0.20	19.4
Validation dataset												
PI&SE (n = 142)	2.68	1.11	41.5	20.9	4.24	20.3	53.8	21.1	39.3	1.02	0.20	19.7
HD (n = 98)	8.96	1.61	18.0	14.2	2.30	16.2	128.6	34.5	26.9	1.08	0.20	18.9
Across all stages (n = 240)	5.24	3.37	64.3	18.2	4.84	26.7	84.3	45.9	54.4	1.04	0.20	19.5

PI&SE: panicle initiation and stem elongation stages; HD: heading stage; SD: standard deviation of the mean; CV: coefficient of variation in %.

### The estimation of NNI using two mechanistic approaches

**Figure 1:**
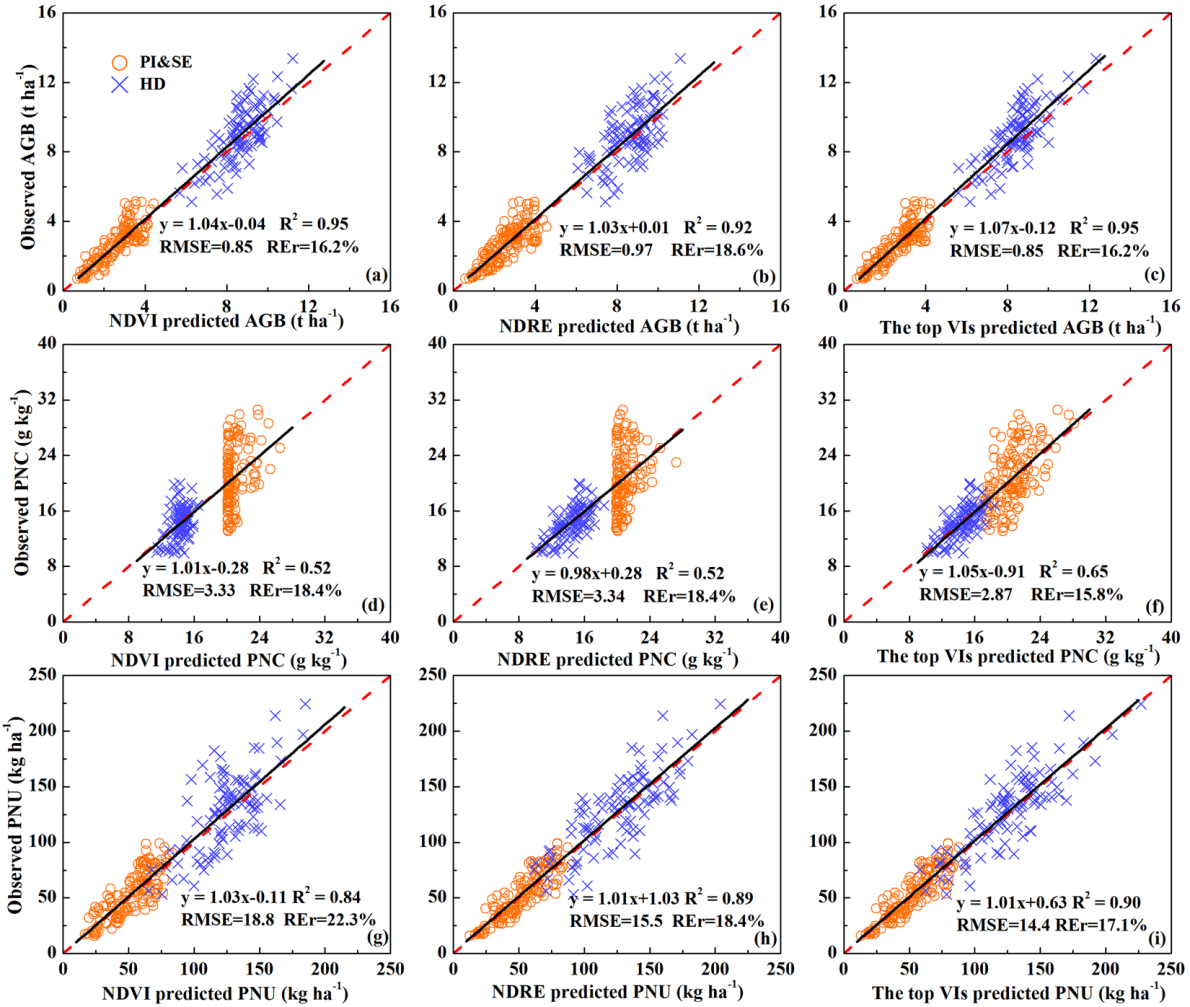
Validation results for the prediction of aboveground biomass (AGB), plant nitrogen concentration (PNC), plant nitrogen uptake (PNU) using the optimal estimation model of NDVI (**a**; **d**; **g**), NDRE (**b**; **e**; **h**) and the top vegetation indices (VIs) (NDVI*RVI and VI_opt_ in c; REPR and REOSAVI in f; REVI_opt_ and NNIRI in i) for specific stages across sites, cultivars, stages and years, respectively. Black lines indicate the regression line and red dotted lines indicate the 1:1 lines.

The performance of NDVI, NDRE and the top VIs to estimate rice AGB, PNC and PNU varied with changed growth stages across different N rate treatments, management systems, cultivars, sites, and years (Table 2). The NDVI explained 76% and 82% of AGB variability at the PI&SE growth stages and across stages, respectively. The corresponding top VIs, NDVI*RVI (77%) at the PI&SE stages and wide dynamic range vegetation index (WDRVI, 86%) across growth stages performed similarly or slightly better, respectively. At the HD stage, however, NDVI explained 53% of AGB variability, while the top VI (VI_opt_) explained 60% of AGB variability. No matter at which growth stage, NDRE did not perform any better than NDVI for AGB estimation. The validation results were similar to the calibration results (Fig. 1a to c). NDVI and the top VIs had equal performance for predicting AGB, with R^2^, root mean square error (RMSE) and relative error (REr) being 0.95, 0.85 and 16.2%, respectively. In comparison, NDRE had similar, but slightly worse performance (R^2^ = 0.92, RMSE = 0.97 and RE = 18.6%) across sites, cultivars, growth stages, and years.

**Table 2:** Coefficient of determination (R^2^) for relationships between NDVI, NDRE and the top vegetation indices calculated from RapidSCAN and aboveground biomass (AGB), plant nitrogen concentration (PNC) and plant nitrogen uptake (PNU) and relationships between vegetation indices and nitrogen sufficiency index (NSI) calculated from vegetation indices and nitrogen nutrition index (NNI) across sites, cultivars, stages and years.

	PI&SE			HD			Across all stages		
	Index	Model	R^2^	Index	Model	R^2^	Index	Model	R^2^
AGB (t ha^−1^)	NDVI	E	0.76	NDVI	P	0.53	NDVI	E	0.82
	NDRE	P	0.70	NDRE	P	0.43	NDRE	E	0.76
	NDVI*RVI	E	0.77	VI_opt_	P	0.60	WDRVI	E	0.86
PNC (g kg^−1^)	NDVI	Q	0.08	NDVI	P	0.20	NDVI	Q	0.32
	NDRE	Q	0.07	NDRE	P	0.55	NDRE	Q	0.16
	REPR	P	0.22	REOSAVI	P	0.55	MEVI	Q	0.39
PNU (kg ha^−1^)	NDVI	E	0.73	NDVI	P	0.51	NDVI	E	0.80
	NDRE	P	0.81	NDRE	P	0.69	NDRE	E	0.87
	REVI_opt_	P	0.81	NNIRI	P	0.74	NNIRI	E	0.88
NNI	NDVI	Q	0.19	NDVI	P	0.36	NDVI	Q	0.18
	NDRE	Q	0.38	NDRE	P	0.68	NDRE	Q	0.37
	RETVI	Q	0.42	REOSAVI	P	0.68	RERVI	Q	0.44
NNI-NSI	NSI_NDVI	Q	0.57	NSI_NDVI	E	0.59	NSI_NDVI	P	0.48
	NSI_NDRE	E	0.58	NSI_NDRE	P	0.70	NSI_NDRE	E	0.61
	NSI_NNIRI	Q	0.61	NSI_NNIRI	P	0.70	NSI_RERVI	P	0.63

PI&SE: panicle initiation and stem elongation stage; HD: heading stage; NNI-NSI: using nitrogen sufficiency index (NSI) calculated from vegetation indices to estimate nitrogen nutrition index (NNI); Q, E and P: the quadratic, exponential, and power fit.

Three RE-based indices, RE point reflectance (REPR), RE optimal soil adjusted VI (REOSAVI), and modified enhanced VI (MEVI), were chosen as the top VIs to estimate PNC. At the PI&SE growth stages and across growth stages, the association between all VIs and PNC were poor (R^2^ < 0.39). At the HD growth stage, REOSAVI and NDRE were moderately related to PNC (R^2^ = 0.55). NDVI did not perform well at any of the growth stages (R^2^ < 0.32). Our validation results confirmed the top VIs (REPR and REOSAVI) had the best performance for the prediction of PNC, with R^2^, RMSE and REr being 0.65, 2.87 and 15.8%, respectively (Fig. 1f). Although there was a good validation result at the HD stage, NDRE did not perform well at PI&SE stage, and as a result, the overall performance was similar to NDVI (R^2^ = 0.52 and REr = 18.4%) across all growth stages (Fig. 1d,e).

The top VIs, optimized RE vegetation index (REVI_opt_) at the PI&SE stages and normalized NIR index (NNIRI) at the HD stage and across all stages, were all RE-based VIs, and they had the best performance for estimating PNU (R^2^ = 0.74–0.88). NDRE (R^2^ = 0.69–0.87) performed similarly well as the top VIs. Comparatively, NDVI explained less PNU variability, especially at the HD stage (R^2^ = 0.51). The validation results indicated that NDRE (R^2^ = 0.89) also had comparable performance as the top VIs (R^2^ = 0.90), which were all better than NDVI (R^2^ = 0.84) (Fig. 1g–i).

**Figure 2:**
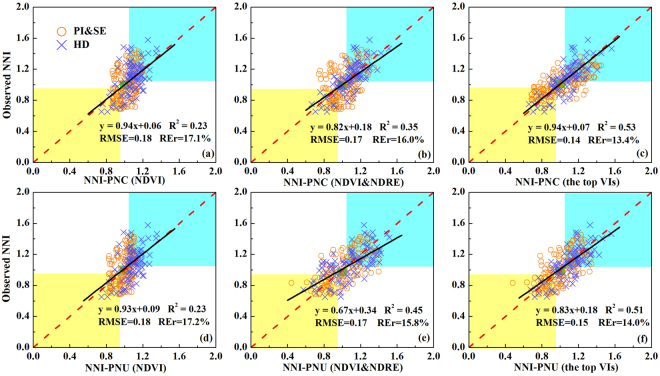
Validation results of nitrogen nutrition index (NNI) for two different mechanistic nitrogen status diagnostic approaches (NNI-PNC in a, b and c; NNI-PNU in **d,e** and **f**) using the optimal estimation model of NDVI (**a,d**), NDVI&NDRE (**b,e**) and the top vegetation indices (VIs) (**c,f**) for specific stages across sites, cultivars, stages and years. NDVI&NDRE is using the optimal estimation model of specific stages of NDVI and NDRE to estimate aboveground biomass (AGB) and plant nitrogen concentration (PNC) or plant nitrogen uptake (PNU), respectively. The top VIs are using all of the top vegetation indices in Fig. 1 to estimate AGB, PNC and PNU at specific stages, respectively. Black lines indicate the regression line and red dotted lines indicate the 1:1 lines. The data in three color areas indicate correctly diagnosed rice nitrogen status: yellow (N deficient), green (N optimal), blue (N surplus).

Based on above results, two different mechanistic approaches (NNI-PNC and NNI-PNU) (see Methods formulas (1), (2) and (3)) were used in this study to estimate NNI and the validation results were shown in Fig. 2. Except that the NNI-PNU method using NDRE (R^2^ = 0.45) provided better results than those of NNI-PNC method (R^2^ = 0.35), there was no significant difference between these two approaches when using NDVI or the top VIs across all stages. No matter which approach was used, the top VI performed the best (R^2^ = 0.51–0.53, RMSE = 0.14–0.15 and REr = 13.4–14.0%), followed by NDVI&NDRE (R^2^ = 0.35–0.45, RMSE = 0.17 and REr = 15.8–16.0%), and the worst was NDVI (R^2^ = 0.23, RMSE = 0.18 and RE = 17.1–17.2%).

### The estimation of NNI using two semi-empirical approaches

The relationships between VIs and NSI calculated from VIs (NSI_VIs) and NNI across sites, cultivars, growth stages, and years were listed in Table 2. The RE- based VIs that had the best correlations with NNI were also chosen as the top VIs. They were RE transformed vegetation index (RETVI), REOSAVI, RE ratio vegetation index (RERVI), NSI_NNIRI and NSI_RERVI at specific growth stages or across all stages.

At the PI&SE stages and across all stages, the correlations of all VIs with NNI were not satisfactory (R^2^ < 0.44). At the HD stage, REOSAVI and NDRE explained 68% of NNI variability. Compared with the VIs, NSI_VIs were more strongly related to NNI. The performance of NSI_VIs varied with growth stages. At the PI&SE stages and across all stages, the NSI_VIs (R^2^ = 0.48–0.63) performed much better than their corresponding original VIs (R^2^ = 0.18–0.44). There was no significant difference among NSI_NDVI, NSI_NDRE and NSI_NNIRI at the PI&SE stages, whereas across all stages NSI_NDRE and NSI_RERVI (R^2^ = 0.61–0.63) performed much better than NSI_NDVI (R^2^ = 0.48). At the HD stage, NSI_NDVI explained 23% more variability in NNI than NDVI, while the improvements of NSI-NDRE and NSI_NNIR were small (Table 2).

**Figure 3:**
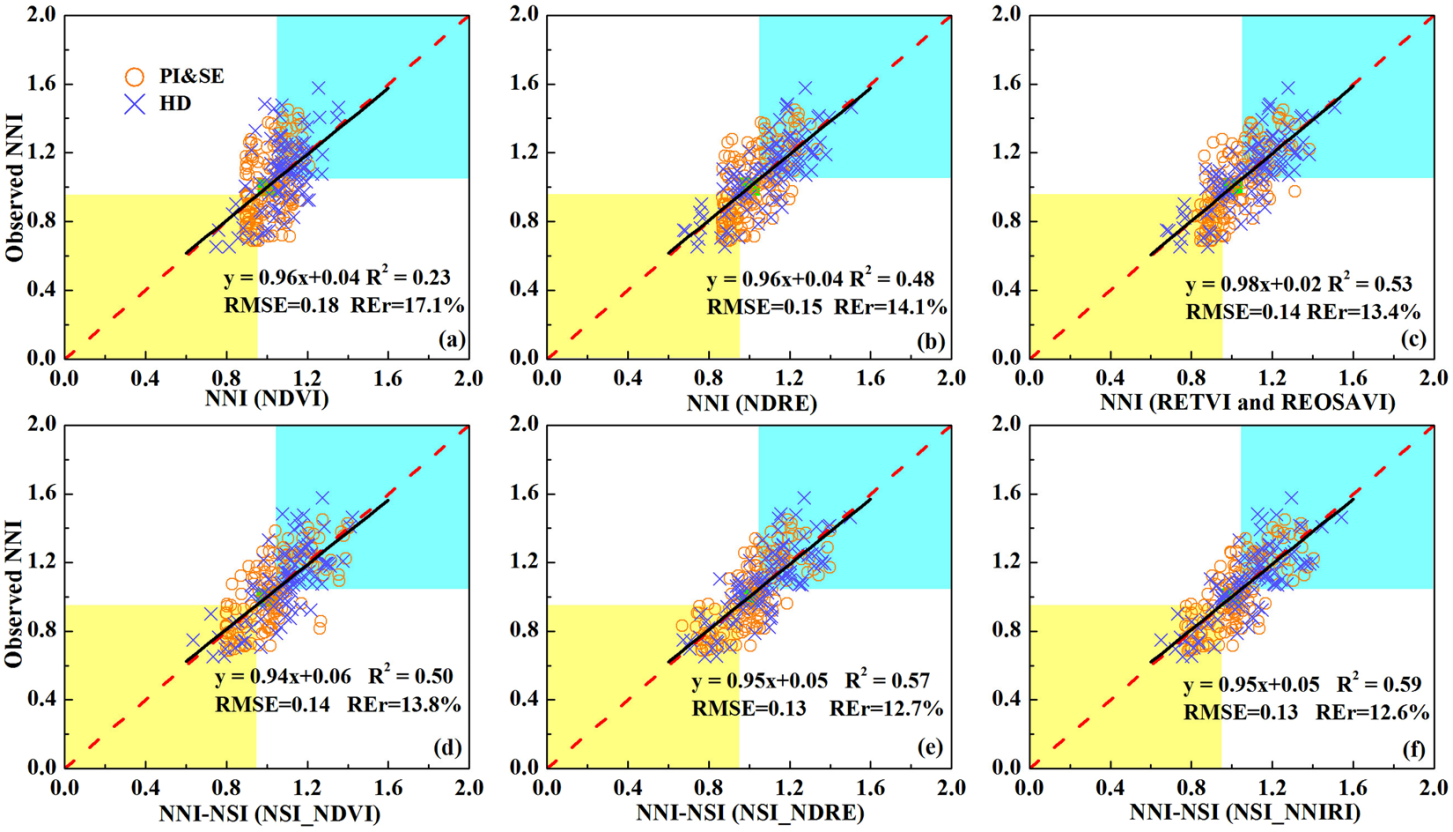
Validation results of nitrogen nutrition index (NNI) for two different semi-empirical nitrogen status diagnostic approaches (NNI in **a,b** and **c**; NNI-NSI in **d,e** and **f**) using the optimal estimation model of NDVI (**a**), NSI_NDVI (**d**), NDRE (**b**), NSI_NDRE (**e**), the top vegetation indices (VIs) (RETVI and REOSAVI in **c**) and the top nitrogen sufficiency index (NSI) calculated from vegetation indices (NSI_NNIRI in **f**) for specific stages across sites, cultivars, stages and years. Black lines indicate the regression line and red dotted lines indicate the 1:1 lines. The data in three color areas indicate correctly diagnosed rice N status: yellow (N deficient), green (N optimal), blue (N surplus).

The validation results of NNI for these two different semi-empirical approaches (NNI-VI and NNI-NSI-VI) using the optimal estimation model of a NDVI, NSI_NDVI, NDRE, NSI_NDRE, the top VIs (RETVI and REOSAVI) and the top NSI-VI (NSI_NNIRI) also confirmed this observation (Fig. 3). For the NNI-VI approach, the validation results of RETVI and REOSAVI were the best (R^2^ = 0.53, RMSE = 0.14 and REr = 13.4%), closely followed by NDRE (R^2^ = 0.48, RMSE = 0.15 and REr = 14.1%), with the NDVI (R^2^ = 0.23, RMSE = 0.18 and REr = 17.1%) being the worst. For the NNI-NSI-VI approach, there was little difference between NSI_NDRE and NSI_NNIRI, but they could explain 7% and 9% more variability in NNI than NSI_NDVI, respectively. Except that the top NSI-VI explained slightly more (6%) variability than the top VIs, NSI_NDVI and NSI_NDRE explained 27% and 9% more variability in NNI than NDVI and NDRE, respectively (Fig. 3).

### Evaluating different nitrogen status diagnostic approaches

**Figure 4:**
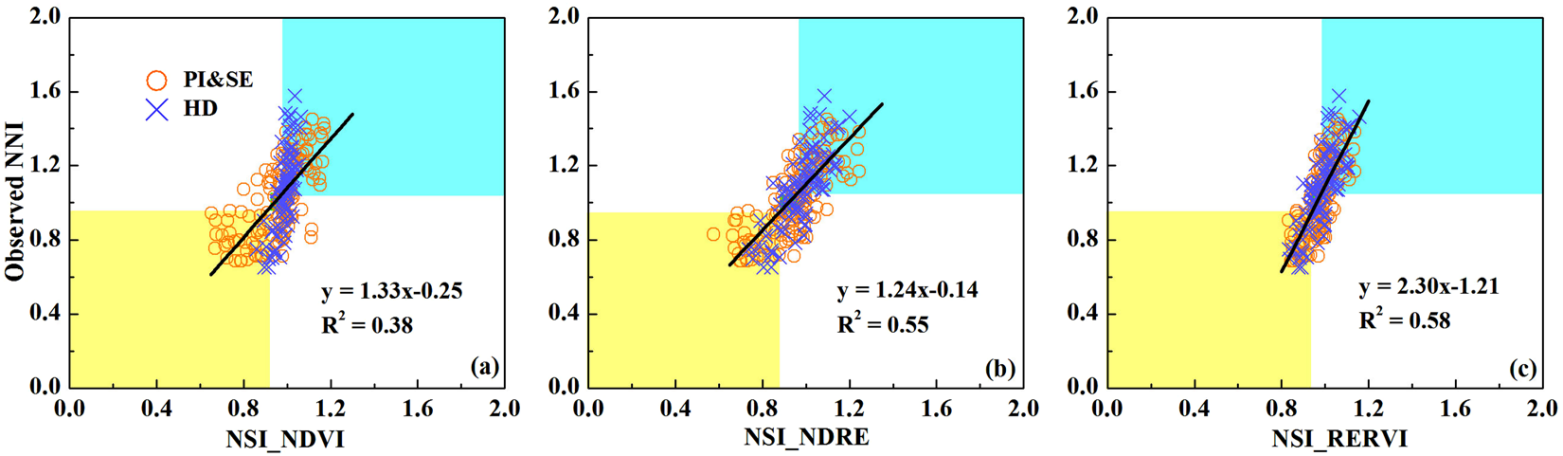
The relationships between nitrogen nutrition index (NNI) and NSI_NDVI (**a**), NSI_NDRE (**b**) and the top nitrogen sufficiency index (NSI) calculated from vegetation indices (NSI_RERVI in **c**) for different growth stages across sites, cultivars and years. Black lines indicate the regression line. The data in three color areas indicate correctly diagnosed rice N status: yellow (N deficient), green (N optimal), blue (N surplus). That is, the yellow, green and blue area mean N deficient, N optimal and N surplus, respectively.

To evaluate the diagnostic accuracy of five different approaches, the validation dataset was divided into three classes: N deficient, N optimal and N surplus based on destructively measured NNI and the threshold values proposed in this study (Methods). The diagnosis results of five different approaches were compared with the results based on measured NNI and the correct diagnosis results were in yellow (N deficient), green (N optimal) and blue (N surplus) areas of Figs 2 to 4. Areal agreement and kappa statistics for five different N status diagnostic approaches were listed in Table 3. The strength of the agreement was fair, moderate and substantial if the kappa statistics was 0.21–0.40, 0.41–0.60 and 0.61–0.80, respectively^30^.

**Table 3:** Areal agreement and kappa statistics for different N status diagnostic approaches across sites, cultivars, stages and years.

Index	Approach	Areal (%)	agreement	Kappa statistics	
		PI&SE	HD	PI&SE	HD
NDVI	NNI-PNC	41	63	NS	0.30
	NNI-PNU	44	63	0.18	0.30
	NNI (NDVI)	44	63	0.14	0.30
	NNI-NSI (NSI_NDVI)	59	74	0.38	0.51
	NSI_NDVI	58	67	0.33	0.38
NDVI&NDRE	NNI-PNC	46	74	0.15	0.52
	NNI-PNU	63	66	0.41	0.39
	NNI (NDRE)	55	76	0.30	0.54
	NNI-NSI (NSI_NDRE)	61	74	0.40	0.53
	NSI_NDRE	59	72	0.38	0.52
The top VIs	NNI-PNC	63	74	0.41	0.54
	NNI-PNU	63	76	0.41	0.54
	NNI (RETVI and REOSAVI)	63	74	0.42	0.53
	NNI-NSI (NSI_NNIRI)	60	76	0.39	0.56
	NSI_RERVI	59	71	0.38	0.51

PI&SE: panicle initiation and stem elongation stage; HD: heading stage; NDVI&NDRE: using the optimal estimation model of specific stages of NDVI and NDRE to estimate aboveground biomass (AGB) and plant nitrogen concentration (PNC) or plant nitrogen uptake (PNU), respectively; NS: no significance at p > 0.05.

The five approaches of the top VIs represent the best potential of using the RapidSCAN sensor for rice N status diagnosis. Their diagnosis accuracies ranged from 59% to 76%, with kappa statistics being 0.38–0.56. At the PI&SE stages, the two mechanistic approaches and the semi-empirical method of using VI to estimate NNI directly achieved the same diagnostic accuracy (63%). Using both NDVI and NDRE, the NNI-PNU approach also achieved this accuracy level, while no approaches using only NDVI achieved the same accuracy. At the HD stage, all the approaches using the top VIs achieved a diagnostic accuracy rate of over 70%, with the NNI-PNU and NNI-NSI approaches being the best (76%) (Table 3). Similar results were achieved using both NDVI&NDRE, except for the NNI-PNU approach. For the approaches using only NDVI, only the NNI-NSI approach achieved an accuracy rate of over 70%.

These results indicated that in general, the diagnostic accuracy was higher at the HD stage than the PI&SE stages. The NNI-NSI approach was the most stable approach, followed by the NSI approach, which was less influenced by the VIs used. The NNI-PNU approach using NDVI&NDRE (63%) and the NNI-VI approach using NDRE (76%) could achieve the same accuracy rate as using top VIs at the PI&SE and HD stages, respectively. Only using the original NDVI index, none of the two mechanistic approaches and the NNI-VI semi-empirical approach performed satisfactorily (Table 3).

## Discussion

Before wider application of the RapidSCAN sensor, several questions need to be answered: 1) How accurate can this sensor be when used for rice N status diagnosis at different growth stages? 2) Will these two default VIs be good enough for rice N status diagnosis at key growth stages? 3) Which NNI estimation approach should be implemented when using this sensor for rice N status diagnosis? 4) Should the suitable VI and NNI estimation approach change with different growth stages? This research was conducted in order to address these questions.

The results of this study indicated that the highest diagnostic accuracy obtained with the RapidSCAN sensor differed with growth stages, being 63% and 76% at the PI&SE and HD growth stages, respectively. No similar study has been conducted with rice. Therefore, this can’t be compared with previous results. For spring maize, the highest N status diagnostic accuracies obtained using the GreenSeeker sensor were 81% and 71% at the V7-V8 and V9-V10 growth stages, respectively^10^.

The two default indices (NDVI and NDRE) are good enough for rice N status diagnosis without the need of other indices. Although using the top VIs (R^2^ = 0.65) significantly improved the prediction of PNC compared with using NDVI and NDRE (R^2^ = 0.52), as well as some improvements in the prediction of NNI (R^2^ = 0.51–0.59 vs. R^2^ = 0.23–0.57), using NDVI and NDRE together could achieve the same N status diagnostic accuracy (63% and 76% at the PI&SE and HD stages, respectively) as using the top VIs. This is an encouraging finding, which will make it easier for producers to use this sensor.

Which approach of N status diagnosis is most accurate and stable? The two mechanistic approaches and the semi-empirical approach (NNI-VI) performed similarly when NDVI (areal agreement = 41–63%) or the top VIs (areal agreement = 63–76%) were used. Xia *et al*. found similar results when using NDVI for diagnosing maize N status (areal agreement = 67–71% for the three approaches)^10^. However, when both NDVI and NDRE were used, the mechanistic NNI-PNU approach performed the best at the PI-SE stage, but the semi-empirical NNI (NDRE) method was the best at the HD stage. Such results indicated that the performance of these three different approaches was dependant on the VIs used and the growth stages or databases. By comparing the mechanistic approach (NNI-PNC) and semi-empirical approach (NNI-VI) for estimating winter wheat NNI, the mechanistic approach was found to be more stable across growth stages, while the semi-empirical approach was more influenced by growth stages^24^. This is supported by the fact that NNI is a relative value not significantly influenced by growth stages while most of VIs are significantly related to AGB and PNU, which increase with growth stages. Therefore, growth stage-specific models will be needed using the semi-empirical NNI-VI approach^25^. A promising approach to overcome the influence of growth stages is to use the NSI or RI approach, which uses a N rich plot or strip as a reference to normalize the VIs^10,27^. Xia *et al*. found that using NDVI to calculate RI and then estimate NNI for diagnosing maize N status was similar to or more accurate and stable (areal agreement = 71–76%) than the mechanistic approaches and the NNI-VI semi-empirical approach^10^. The results in this study indicated that the NNI-NSI approach was a very stable approach, without being influenced significantly by the VIs used. Its advantage was most obvious when only NDVI was used (areal agreement = 59–74% vs. 41–63%). This NNI-NSI approach using either NDVI or NDRE could achieve similar performance as approaches using top VIs. The fifth approach (NSI-VI) using NSI to diagnose N status directly was also quite stable without being influenced much by the VIs used. It was the simplest approach, but it was slightly less accurate than the NNI-NSI approach. This result was different from Xia *et al*. who found that the NSI_NDVI approach performed the best among all the five approaches evaluated^10^. Their studies only involved one maize cultivar, while this study involved two rice cultivars. The crops were also very different. Their study only considered the early growth stages (V7-V10), while this study also considered a later stage (the HD stage). Therefore, more studies are needed to further evaluate the fifth approach for rice. As a result, the NNI-NSI approach should be recommended for rice N status diagnosis, without the need to change the VIs used at different growth stages.

In production agriculture, we not only need to diagnose rice N status, but also need to make N topdressing recommendations^22^. After the rice N status was diagnosed as N surplus, optimum or deficient, a fixed amount of N fertilizers can be decreased or increased on the basis of planned N topdressing rate, as using the chlorophyll meter for N recommendation^31^. The top dressing N fertilizer rate can also be adjusted according to the estimated NNI, which can indicate the magnitude of N surplus or deficiency. A more quantitative approach is to calculate the difference between PNU and critical PNU, which can be used to adjust topdressing N application rates^22^. If so, the mechanistic NNI-PNU approach should be recommended for NNI estimation, because in general topdressing N is applied at the SE stage for rice in Northeast China, and the NNI-PNU approach using NDVI and NDRE performed the same as approaches using top VIs. Several other active crop sensor-based N recommendation algorithms not related to NNI estimation have also been developed^8,11,32^, which should also be evaluated using the RapidSCAN sensor. More studies are needed to further evaluate the NNI-NSI and NNI-PNU approaches to using the RapidSCAN sensor for rice N status diagnosis under diverse on-farm conditions and develop sensor-based in-season N recommendation algorithms. Such sensor-based PNM strategies should also be integrated with high yield crop management practices for increasing both crop yield and N use efficiency^33^.

## Methods

### Study site

The study was conducted in Sanjiang Plain, Heilongjiang Province, Northeast China (47.2°N, 132.6°E). The main soil type in this area is Albic soil, classified as Mollic Planosols in the FAO-UNESCO system, and typical Argialbolls in the Soil Taxonomy. The study site possesses a cool-temperate sub-humid continental monsoon climate. The temperature ranges from −41 °C in the winter to 38 °C in the summer, with a mean annual temperature of 1.9 °C. About 72% of its annual precipitation (500–600 mm) occurs from June to September. The frost-free period is only about 120–140 days long yearly^33^.

### Plot and on-farm experiments for calibration and validation

Sixteen plot experiments were conducted from 2014 to 2016 at Jiansanjiang Experiment Station of the China Agricultural University, involving two different cultivars, different N rates, sensor-based N management strategies, and precision rice management strategies (Table S1). Six N rate experiments (EXP. 1–6) were conducted from 2014 to 2016. Each experiment had the same five N rates (0, 40, 80, 120 and 160 kg N ha^−1^). Exp 1 to 3 used Longjing 31 rice cultivar, which is an 11-leaf cultivar requiring 130 days to reach maturity. Exp 4 to 6 used Longjing 21 rice cultivar, which is a 12-leaf cultivar that needs about 133 days to maturity. Another five field experiments (Exp. 7 to 12) were conducted from 2014 to 2016. Each experiment consisted of the same four active canopy sensor-based precision N management strategy treatments: three active canopy sensor-based precision N management treatments using the GreenSeeker, Crop Circle ACS-470, and the RapidSCAN sensors, respectively. The basal N rate was 48 kg ha^−1^ before transplanting, and the tiller fertilizer rate was 36 kg ha^−1^ at the tillering stage for all these three treatments. The panicle fertilizer rate was determined according to different active canopy sensor-based N recommendation algorithms and applied at the stem elongation stage. The fourth treatment applied 84 kg ha^−1^ as basal N fertilizer in the form of slow release urea (44% N) without applying any tiller fertilizer, and the panicle fertilizer was based on active canopy sensor-based recommendation. The crop cultivars for Exp. 7 to 9 and Exp. 10 to 12 were Longjing 31 and Longjing 21 rice cultivars, respectively. In addition, another four field experiments (Exp.13 to 16) were conducted from 2015 to 2016. Each experiment consisted of the same seven rice management strategy treatments: (i) the control treatment, with no N application, (ii) farmer’s practice, with 150 kg N ha^−1^ as total N rates, (iii) regional optimum rice management, with 110 kg N ha^−1^ as total N rates, (iv) GreenSeeker-based precision rice management, (v) Crop Circle-based precision rice management strategy 1, (vi) Crop Circle-based precision rice management strategy 2, and (vii) Crop Circle-based precision rice management strategy 3. The total N rates of the treatment iv to vii were different according to active crop sensor-based N recommendation strategies. The rice cultivars for Exp. 13 to 14 and Exp. 15 to 16 were Longjing 31 and Longjing 21, respectively. All plot experiments were replicated three times in a randomized complete block design. The N source was granular urea (except the slow-release urea treatment). For all the treatments, 50 kg P_2_O_5_ ha^−1^ in the form of Ca(H_2_PO_4_)_2_ was applied before transplanting and 105 kg K_2_O ha^−1^ in the form of potassium chloride was applied as two splits: 50% before transplanting and 50% at the stem elongation (SE) stage. Rice seedlings were prepared in a greenhouse and transplanted into the experimental fields.

In addition to the aforementioned sixteen plot experiments, ten on-farm experiments were conducted in cooperation with farmers (five fields in 2015 and 2016, respectively) in two villages of Qixing Farm in Jiansanjiang, Heilongjiang Province, under on-farm conditions (Table S1). Treatments in each experiment were three rice management strategies including farmer’s practice, regional optimum rice management, and active canopy sensor-based or UAV-based precision rice management. The rice cultivars included Longjing 31 and Longjing 46 (an 11-leaf cultivar that requires 127 days to reach maturity).

### RapidSCAN-based remote sensing data collection

RapidSCAN (Holland Scientific Inc., Lincoln, Nebraska, USA) is a portable active crop canopy sensor that integrates a data logger, graphical display, GPS, active crop sensor and power source into a small and compact instrument. The sensor incorporates red (R; 670 nm), red-edge (RE; 730 nm), and near infra-red (NIR; 780 nm) spectral bands. Furthermore, the sensor is capable of collecting data from vegetation at sensor-to-canopy distances ranging from 0.3 m to 3 m and is not affected by measurement height within that range. The sensor was held about 0.7–0.9 m above rice canopy and carried at a consistent speed to collect sensors’ readings from four rows in each plot. The reflectance values were then averaged to represent reflectance for each plot. Fifty-one vegetation indices (VIs) evaluated in this study are listed in Table S2.

### Plant sampling and measurement

Rice plant samples were acquired right after collecting sensing data at the panicle initiation (PI) and SE growth stage and at the heading (HD) growth stage. On the measurement dates, destructive plant samples of above ground biomass (AGB) were taken by randomly clipping three hills from the scanned plants according to the average tillering numbers in each plot. All samples were rinsed with water, and the roots were removed. The samples were separated into leaves and stems, which were put into oven for deactivation of enzymes under 105 °C for half an hour, and then dried at 80 °C until constant weight and weighed to determine AGB. Sub-samples that passed through 1 mm screen in a dry sample mill were mineralized using H_2_SO_4_–H_2_O_2_, and PNC was determined using the Kjeldahl-N method. The PNU was determined by multiplying PNC with AGB.

The critical N dilution curve of rice in Northeast China developed by Huang *et al*.^34^ shown in Equation (1) was used in this study:

1 $${{\rm{N}}}_{{\rm{c}}}=27.7\,{{\rm{W}}}^{-0.34}$$

where Nc is the critical N concentration expressed as g kg^−1^ dry matter (DM) and W is the AGB expressed in t DM ha^−1^.

The NNI was calculated following Equation (2)^19^:

2 $${\rm{NNI}}={{\rm{N}}}_{{\rm{a}}}/{{\rm{N}}}_{{\rm{c}}}$$

where N_a_ is the actual measured N concentration and N_c_ is the critical N concentration as determined by Equation (1).

The NNI can also be calculated using PNU, as shown in Equation (3):

3 $${\rm{NNI}}={{\rm{PNU}}}_{{\rm{a}}}/{{\rm{PNU}}}_{{\rm{c}}}$$

where PNU_a_ is the actual measured PNU and PNU_c_ is the critical PNU (N_c_ × AGB).

According to Huang *et al*.^22^ and practical applications, we classified the rice N status into three categories based on NNI values: deficient N status (NNI < 0.95), optimal N status (0.95 ≤ NNI ≤ 1.05) and surplus N status (NNI > 1.05).

### Different approaches to estimate NNI

To evaluate the possibility of using normalized VIs to improve the estimation of NNI, Nitrogen Sufficiency Index (NSI) was calculated in this study. NSI is the sensor data from the field to be fertilized divided by averaged sensor data from the well-fertilized reference plots^27,35^. RapidSCAN vegetation indices were used to calculate NSI in the NSI_VI method. In this study, the plots of 160 kg N ha^−1^ and farmer’s practice in on-farm experiments with sufficient N supply were used as N rich plots, and their average VI values were used for NSI calculation.

As we mentioned in the Introduction section, there are five different approaches to be taken to non-destructively diagnose N status with the RapidSCAN sensor. Specifically, the first approach is to estimate PNC using NDVI, NDRE and the top VIs that yield the best results; next, from the estimated AGB, N_c_ can be determined using the established critical N dilution curve^10^ and then NNI can be calculated NNI-PNC (NDVI), NNI-PNC (NDRE) or NNI-PNC (top VIs), respectively. The second approach estimates PNU, rather than PNC, using NDVI, NDRE and the top VIs. The PNU_c_ can be calculated by the estimated AGB and N_c_ obtained by using that AGB and critical N dilution curve. Then NNI can be calculated as (NNI-PNU (NDVI), NNI-PNU (NDRE) or NNI-PNU (top VIs), respectively). The third approach is to estimate NNI directly using RapidSCAN NDVI (NNI (NDVI)), NDRE (NNI (NDRE)) and the top VIs (NNI (RETVI and REOSAVI)). The fourth approach is to estimate NNI directly using NSI calculated with RapidSCAN NDVI (NNI-NSI_NDVI), NDRE (NNI-NSI _NDRE) and the top VIs (NNI-NSI_NNIRI). The fifth approach is to use the relationship between NSI and NNI to determine the corresponding NSI threshold values across sites, cultivars, growth stages, and years. In this study, we used NSI_NDVI, NSI_NDRE and NSI_RERVI (the top performing NSI_VIs to estimate NNI across all stages) to directly diagnose rice N status. The NSI threshold values to diagnose rice N status directly were determined as follows: deficient N status (NSI_NDVI < 0.92, NSI_NDRE < 0.89 or NSI_RERVI < 0.94), surplus N status (NSI_NDVI > 0.98, NSI_NDRE > 0.98 or NSI_RERVI > 0.99) and intermediate values for optimal N status.

### Statistical analysis

Data collected from the plot experiments and on-farm experiments were pooled together and then randomly divided into calibration dataset (67% of the observations) and validation dataset (33% of the observations). The mean value, standard deviation (SD) and coefficient of variation (CV, %) of rice agronomic parameters were calculated using Microsoft Excel (Microsoft Corporation, Redmond, Washington, USA). The coefficients of determination (R^2^) of the relationships between VIs and agronomic parameters were calculated using SPSS 18.0 (SPSS Inc., Chicago, Illinois, USA), and the model with the highest R^2^ was selected. In addition to R^2^, the performance of the model for predicting rice status indicators was evaluated using the root mean square error (RMSE) and the relative error (REr).

Different N status diagnostic approaches were evaluated by areal agreement and kappa statistics^36^. The areal agreement is the percentage of plots that shared a common classification (N deficient, N optimal and N surplus), whereas the Kappa statistics provides a more robust measure of how two classifications agreed compared with a “chance” agreement.

### Data availability

The datasets generated during and/or analyzed during the current study are available from the corresponding author on reasonable request.

## Electronic supplementary material

Supplementary Information
