# Supplementary material for: Evaluating different approaches to non-destructive nitrogen status diagnosis of rice using portable RapidSCAN active canopy sensor
Source: Sci Rep. 2017 Oct 26;7:14073. doi: 10.1038/s41598-017-14597-1 (PMC5658326; doi:10.1038/s41598-017-14597-1)
Supplement: Supplementary file 1 — Supplementary Information [file 41598_2017_14597_MOESM1_ESM.pdf]

Evaluating different approaches to non-destructive nitrogen status diagnosis of rice using portable RapidSCAN active canopy sensor

Junjun Lu[1], Yuxin Miao[1*], Wei Shi[1], Jingxin Li[1], and Fei Yuan[2]

[1] International Center for Agro-Informatics and Sustainable Development, College of Resources and Environmental Sciences, China Agricultural University, Beijing 100193, China;
[2] Department of Geography, Minnesota State University, Mankato, MN, 56001, USA
***Corresponding Author: Yuxin Miao**

ymiao@cau.edu.cn; ymiao2007@gmail.com

Table S1. Detailed information about the experiments conducted in this study.

Experiment		Year	Cultivar	Planting date	Harvest date	Sensing stages	Sensing date
Plot experiment							
Exp. 1	N rate experiments	2014	Longjing 31	19 May	29 Sep.	PI, SE, HD	23 Jun., 3 Jul., 26 Jul.
Exp. 2		2015	Longjing 31	20 May	4 Oct.	PI, SE, HD	24 Jun., 6 Jul. 30 Jul.
Exp. 3		2016	Longjing 31	19 May	25 Sep.	PI, SE, HD	28 Jun., 6 Jul., 25 Jul.
Exp. 4		2014	Longjing 21	19 May	29 Sep.	PI, SE, HD	26 Jun., 7 Jul., 26 Jul.
Exp. 5		2015	Longjing 21	20 May	4 Oct.	PI, SE, HD	24 Jun., 7 Jul., 2 Aug.
Exp. 6		2016	Longjing 21	19 May	25 Sep.	PI, SE, HD	28 Jun., 6 Jul., 25 Jul.
Exp. 7	Active canopy sensor-based precision N management comparison	2014	Longjing 31	19 May	29 Sep.	SE	3 Jul.
Exp. 8		2015	Longjing 31	20 May	4 Oct.	SE	6 Jul.
Exp. 9		2016	Longjing 31	19 May	25 Sep.	SE	6 Jul.
Exp. 10		2014	Longjing 21	19 May	29 Sep.	SE	7 Jul.
Exp. 11		2015	Longjing 21	20 May	4 Oct.	SE	7 Jul.
Exp. 12		2016	Longjing 21	19 May	25 Sep.	SE	6 Jul.
Exp. 13	Rice management strategy comparison	2015	Longjing 31	21 May	3 Oct.	SE, HD	8 Jul., 27 Jul.
Exp. 14		2016	Longjing 31	18 May	24 Sep.	SE, HD	8 Jul., 31 Jul.
Exp. 15		2015	Longjing 21	21 May	3 Oct.	SE, HD	8 Jul., 2 Aug.
Exp. 16		2016	Longjing 21	18 May	24 Sep.	SE, HD	8 Jul., 31 Jul.
On-farm experiment							
Farm 1		2015	Longjing 31	12 May	28 Sep.	SE, HD	29 Jun., 31 Jul.
Farm 2		2015	Longjing 31	12 May	28 Sep.	SE, HD	29 Jun., 31 Jul.

Farm 3	2015	Longjing 31	10 May	28 Sep.	SE	29 Jun.
Farm 4	2015	Longjing 31	12 May	28 Sep.	HD	1 Aug.
Farm 5	2015	Longjing 31	11 May	28 Sep.	HD	1 Aug.
Farm 6	2016	Longjing 46	16 May	23 Sep.	SE, HD	6 Jul., 1 Aug.
Farm 7	2016	Longjing 46	13 May	22 Sep.	SE, HD	7 Jul., 30 Jul.
Farm 8	2016	Longjing 31	19 May	22 Sep.	SE, HD	7 Jul., 30 Jul.
Farm 9	2016	Longjing 31	23 May	23 Sep.	SE	12 Jul.
Farm 10	2016	Longjing 46	11 May	22 Sep.	HD	1 Aug.

Table S2. Spectral indices used in this study.

Index	Formula	Reference
Normalized difference vegetation index (NDVI)	(NIR-R)/(NIR+R)	(1)
Ratio vegetation index (RVI)	NIR/R	(2)
Difference vegetation index (DVI)	NIR-R	(3)
Renormalized difference vegetation index (RDVI)	(NIR-R)/SQRT(NIR+R)	(4)
wide dynamic range vegetation index (WDRVI)	(a*NIR-R)/(a*NIR+R) (a=0.12)	(5) and (6)
Soil-adjusted vegetation index (SAVI)	1.5*(NIR-R)/(NIR+R+0.5)	(7)
Optimized SAVI (OSAVI)	(1+0.16)* (NIR-R)/(NIR+R+0.16)	(8)
Modified Soil-adjusted vegetation index (MSAVI)	0.5*[2*NIR+1-SQRT((2*NIR+1)^2-8*(NIR-R))]	(9)
Transformed Normalized vegetation index (TNDVI)	SQRT((NIR-R)/(NIR+R)+0.5)	(10)

Index	Formula	Reference
Modified simple ratio (MSR)	(NIR/R-1)/SQRT(NIR/R+1)	(11)
Optimal vegetation index (VI_{opt})	$1.45*((NIR^2+1)/(R+0.45))$	(12)
Red edge point reflectance (REPR)	(R+NIR)/2	(13)
Nonlinear index (NLI)	$(NIR^2-R)/(NIR^2+R)$	(14)
Modified nonlinear index (MNLI)	$1.5*(NIR^2-R)/(NIR^2+R+0.5)$	(15)
NDVI*RVI	$(NIR^2-R)/(NIR+R^2)$	(15)
SAVI*SR	$(NIR^2-R)/[(NIR+R+0.5)*R]$	(15)
Normalized difference red edge (NDRE)	(NIR-RE)/(NIR+RE)	(16)
Red edge ratio vegetation index (RERVI)	NIR/RE	(17)
Red edge difference vegetation index (REDVI)	NIR-RE	(6)
Red edge re-normalized different vegetation index (RERDVI)	(NIR-RE)/SQRT(NIR+RE)	(6)
Red edge wide dynamic range vegetation index (REWDRVI)	(a*NIR-RE)/(a*NIR+RE) (a=0.12)	(6)
Red edge soil adjusted vegetation index (RESAVI)	1.5 * [(NIR-RE)/(NIR+RE+0.5)]	(6)
Red edge optimal soil adjusted vegetation index (REOSAVI)	(1+0.16)(NIR-RE)/(NIR+RE+0.16)	(6)
Modified red edge soil adjusted vegetation index (MRESAVI)	$0.5*[2*NIR+1-SQRT((2*NIR+1)^2-8*(NIR-RE))]$	(6)
Optimized red edge vegetation index ($REVI_{opt}$)	100*(lnNIR-lnRE)	(18)
Red edge chlorophyll index (CI_{RE})	NIR/RE-1	(19)
Modified red edge simple ratio (MSR_RE)	(NIR/RE-1)/SQRT(NIR/RE+1)	(6)

Index	Formula	Reference
Red edge normalized difference vegetation index (RENDVI)	(RE-R)/(RE+R)	(20)
Red edge simple ratio (RESR)	RE/R	(21)
Modified red edge difference vegetation index (MREDVI)	RE-R	This study, modified from (3)
MERIS terrestrial chlorophyll index (MTCI)	(NIR-RE)/(RE-R)	(13)
DATT index (DATT)	(NIR-RE)/(NIR-R)	(22)
Normalized near infrared index (NNIRI)	NIR/(NIR+RE+R)	This study, modified from (23)
Normalized red edge index (NREI)	RE/(NIR+RE+R)	This study, modified from (23)
Normalized red index (NRI)	R/(NIR+RE+R)	This study, modified from (23)
Modified double difference index (MDD)	(NIR-RE)-(RE-R)	This study, modified from (24)
Modified red edge simple ratio (MRESR)	(NIR-R)/(RE-R)	This study, modified from (25)
Modified normalized difference (MND)	(NIR-RE)/(NIR+RE-2R)	This study, modified from (25)
Modified enhanced vegetation index (MEVI)	2.5*(NIR-R)/(NIR+6*R-7.5*RE+1)	This study, modified from (26)
Modified normalized difference red edge (MNDRE)	(NIR-RE+2*R)/(NIR+RE-2*R)	This study, modified from (27)
Red edge transformed vegetation index (RETVI)	0.5*[120*(NIR-R)-200*(RE-R)]	This study, modified from (6)
Modified chlorophyll absorption in reflectance index1 (MCARI1)	[(NIR-RE)-0.2*(NIR-R)](NIR/RE)	This study, modified from (6)
Modified chlorophyll absorption in reflectance index 2 (MCARI2)	$\frac{1.5[2.5(NIR-R)-1.3(NIR-RE)]}{\sqrt{(2NIR+1)^2-(6NIR-5\sqrt{R})-0.5}}$	This study, modified from (28)

Index	Formula	Reference
Modified transformed chlorophyll absorption in reflectance index (MTCARI)	3*[(NIR-RE)-0.2*(NIR-R)(NIR/RE)]	This study, modified from (6)
Modified red edge transformed vegetation index (MRETVI)	1.2*[1.2*(NIR-R)-2.5*(RE-R)]	This study, modified from (6)
Modified canopy chlorophyll content index (MCCCI)	NDRE/NDVI	(29)
MCARI1/OSAVI	MCARI1/OSAVI	This study, modified from (30)
MCARI2/OSAVI	MCARI2/OSAVI	This study, modified from (6)
MTCARI/OSAVI	MTCARI/OSAVI	This study, modified from (31)
MCARI1/MRETVI	MCARI1/MRETVI	This study, modified from (32)
MTCARI/MRETVI	MTCARI/MRETVI	This study, modified from (6)

NIR: near infrared band; RE: red-edge band; R: red band.

Extended reference list for spectral indices collection, including 32 references with 51 vegetation indices

1. Rouse, J. W., Haas, J. R. H., Schell, J. A. & Deering, D. W. *Monitoring vegetation systems in the Great Plains with ERTS* (ed. NASA, Proceedings of Thid Earth Resources Technology Satellite-1 Symposium, NASA special publication) 309–317 (Washington, DC, USA, 1974).
2. Jordan, C. F. Derivation of leaf-area index from quality of light on the forest floor. *Ecology*, 663–666 (1969).
3. Tucker, C. J. Red and photographic infrared linear combinations for monitoring vegetation. *Remote Sens. Environ.* **8**, 127–150 (1979).
4. Roujean, J. L. & Breon, F. M. Estimating PAR absorbed by vegetation from bidirectional reflectance measurements. *Remote Sens. Environ.* **51**, 375–384 (1995).
5. Gitelson, A. A. Wide Dynamic Range Vegetation Index for remote quantification of biophysical characteristics of vegetation. *J. Plant Physiol.* **161**, 165–173 (2004).
6. Cao, Q. *et al*. Non-destructive estimation of rice plant nitrogen status with Crop Circle multispectral active canopy sensor. *Field Crops Res.* **154**, 133–144 (2013).
7. Huete, A. R. A soil-adjusted vegetation index (SAVI). *Remote Sens. Environ.* **25**, 295–309 (1988).
8. Rondeaux, G., Steven, M. & Baret, F. Optimization of soil-adjusted vegetation indices. *Remote Sens. Environ.* **55**, 95–107 (1996).
9. Qi, J., Chehbouni, A., Huete, A., Kerr, Y. & Sorooshian, S. A modified soil adjusted vegetation index. *Remote Sens. Environ.* **48**, 119–126 (1994).
10. Sandham, L. & Zietsman, H. Surface temperature measurement from space: a case study in the south Western Cape of South Africa. *S. Afr. J. Enol. Vitic.* **18**, 25–30 (1997).
11. Chen, J. M. Evaluation of vegetation indices and a modified simple ratio for boreal applications. *Can. J. Remote Sens.* **22**, 229–242 (1996).
12. Reyniers, M., Walvoort, D. J. J. & Baardemaaker, J. D. A linear model to predict with a multi‐spectral radiometer the amount of nitrogen in winter wheat. *Int. J. Remote Sens.* **27**, 4159–4179 (2006).
13. Dash, J. & Curran, P. J. The MERIS terrestrial chlorophyll index. *Int. J. Remote Sens.* **25**, 5403–5413 (2004).
14. Goel, N. S. & Qin, W. Influences of canopy architecture on relationships between various vegetation indices and LAI and FPAR. *Remote Sens. Rev.* **10**, 309–347 (1994).
15. Gong, P., Pu, R., Biging, G. S. & Larrieu, M. R. Estimation of forest leaf area index using vegetation indices derived from Hyperion hyperspectral data. *IEEE T. Geosci. Remote* **41**, 1355–1362 (2003).
16. Barnes, E. M. *et al*. Coincident detection of crop water stress, nitrogen status and canopy density using ground-based multispectral data. *International Conference on Precision Agriculture and Other Resource Management*, 16–19 (2000).

17. Gitelson, A. A., Merzlyak, M. N. & Lichtenthaler, H. K. Detection of red edge position and chlorophyll content by reflectance measurements near 700 nm. *J. Plant Physiol.* **148**, 501–508 (1996).
18. Jasper, J., Reusch, S. & Link, A. *Active sensing of the N status of wheat using optimized wavelength combination: impact of seed rate, variety and growth stage* (ed. Van Henten, E. J., Goense D. & Lokhorst, C., Precision Agriculture 09: Papers from the 7th European Conference on Precision Agriculture. Wageningen Academic Publishers) 23–30 (Wageningen, Netherlands, 2009).
19. Gitelson, A. A., Gritz, Y. & Merzlyak, M. N. Relationships between leaf chlorophyll content and spectral reflectance and algorithms for non-destructive chlorophyll assessment in higher plant leaves. *J. Plant Physiol.* **160**, 271–282 (2003).
20. Elsayed, S., Rischbeck, P. & Schmidhalter, U. Comparing the performance of active and passive reflectance sensors to assess the normalized relative canopy temperature and grain yield of drought-stressed barley cultivars. *Field Crops Res.* **177**, 148–160 (2015).
21. Erdle, K., Mistele, B. & Schmidhalter, U. Comparison of active and passive spectral sensors in discriminating biomass parameters and nitrogen status in wheat cultivars. *Field Crops Res.* **124**, 74–84 (2011).
22. Datt, B. Visible/near infrared reflectance and chlorophyll content in Eucalyptus leaves. *Int. J. Remote Sens.* **20**, 2741–2759 (1999).
23. Sripada, R. P., Heiniger, R. W., White, J. G. & Meijer, A. D. Aerial color infrared photography for determining early in-season nitrogen requirements in corn. *Agron. J.* **98**, 968–977 (2006).
24. Le Maire, G., François, C. & Dufrêne, E. Towards universal broad leaf chlorophyll indices using PROSPECT simulated database and hyperspectral reflectance measurements. *Remote Sens. Environ.* **89**, 1–28 (2004).
25. Sims, D. A. & Gamon, J. A. Relationships between leaf pigment content and spectral reflectance across a wide range of species, leaf structures and developmental stages. *Remote Sens. Environ.* **81**, 337–354 (2002).
26. Justice, C. O. *et al*. The Moderate Resolution Imaging Spectroradiometer (MODIS): land remote sensing for global change research. *IEEE T. Geosci. Remote* **36**, 1228–1249 (1998).
27. Wang, W. *et al*. Estimating leaf nitrogen concentration with three-band vegetation indices in rice and wheat. *Field Crops Res.* **129**, 90–98 (2012).
28. Haboudane, D., Miller, J. R., Pattey, E., Zarco-Tejada, P. J. & Strachan, I. B. Hyperspectral vegetation indices and novel algorithms for predicting green LAI of crop canopies: Modeling and validation in the context of precision agriculture. *Remote Sens. Environ.* **90**, 337–352 (2004).
29. Long, D. S., Eitel, J. U. & Huggins, D. R. Assessing nitrogen status of dryland wheat using the canopy chlorophyll content index. *Crop Manag.* **8** (2009).
30. Zarco-Tejada, P. J., Miller, J. R., Morales, A., Berjón, A. & Agüera, J. Hyperspectral indices and model simulation for chlorophyll estimation in open-canopy tree crops. *Remote Sens. Environ.* **90**, 463–476 (2004).

31. Haboudane, D., Miller, J. R., Tremblay, N., Zarco-Tejada, P. J. & Dextraze, L. Integrated narrow-band vegetation indices for prediction of crop chlorophyll content for application to precision agriculture. *Remote Sens. Environ.* **81**, 416–426 (2002).
32. Eitel, J. U. H., Long, D. S., Gessler, P. E. and Smith, A. M. S. Using in - situ measurements to evaluate the new RapidEye™ satellite series for prediction of wheat nitrogen status. *Int. J. Remote Sens.* **28**, 4183–4190 (2007).
